# Supplementary material for: Role of Repeat Tract Structure and the rs7158733 SNP in Spinocerebellar Ataxia 3
Source: Int J Mol Sci. 2025 Oct 10;26(20):9836. doi: 10.3390/ijms26209836 (PMC12563690; doi:10.3390/ijms26209836)
Supplement: Supplementary file 1 [file ijms-26-09836-s001.zip › ijms-3844017-supplementary.pdf]

Repeat Size	Repeat Tract Sequence	Participant																																										Total
		1	2	3	4	5	6	7	8	9	10	11	12	13	14	15	16	17	18	19	20	21	22	23	24	25	26	27	28	29	30	31	32	33	34	35	36	37	38	39	40	41	42	43	44	
13	(CAG)$_{2}$(CAA)(AAG)(CAG)(CAA)(CAG)$_{7}$																			1																										1
14	(CAG)$_{2}$(CAA)(AAG)(CAG)(CAA)(CAG)$_{8}$														4			2		4		3																								13
18	(CAG)$_{2}$(CAA)(AAG)(CAG)(CAA)(CAG)$_{12}$																																	1												1
19	(CAG)$_{2}$(CAA)(AAG)(CAG)(CAA)(CAG)$_{13}$		1																										2																		3
21	(CAG)$_{2}$(CAA)(AAG)(CAG)(CAA)(CAG)$_{15}$	1								1		1	1								2																		1								7
22	(CAG)$_{2}$(CAA)(AAG)(CAG)(CAA)(CAG)$_{16}$	1										4	4						1												1													1				12
23	(CAG)$_{2}$(CAA)(AAG)(CAG)(CAA)(CAG)$_{17}$	7				2				5									5				1	1	4		3			3											2					33
24	(CAG)$_{2}$(CAA)(AAG)(CAG)(CAA)(CAG)$_{18}$				6	8																																				3						17
25	(CAG)$_{2}$(CAA)(AAG)(CAG)(CAA)(CAG)$_{19}$						1																																								1
27	(CAG)$_{2}$(CAA)(AAG)(CAG)(CAA)(CAG)$_{21}$						5				5																									2										12
28	(CAG)$_{2}$(CAA)(AAG)(CAG)(CAA)(CAG)$_{22}$																									1																				1
29	(CAG)$_{2}$(CAA)(AAG)(CAG)(CAA)(CAG)$_{23}$								5							1										1																				7
30	(CAG)$_{2}$(CAA)(AAG)(CAG)(CAA)(CAG)$_{24}$							5								6																											1			12
31	(CAG)$_{2}$(CAA)(AAG)(CAG)(CAA)(CAG)$_{25}$		1																																										1
32	(CAG)$_{2}$(CAA)(AAG)(CAG)(CAA)(CAG)$_{26}$		2	3																																									5
33	(CAG)$_{2}$(CAA)(AAG)(CAG)(CAA)(CAG)$_{27}$		3	9										1																		1												1	2	17
34	(CAG)$_{2}$(CAA)(AAG)(CAG)(CAA)(CAG)$_{28}$		1																				1																					2
35	(CAG)$_{2}$(CAA)(AAG)(CAG)(CAA)(CAG)$_{29}$													1																															1
36	(CAG)$_{2}$(CAA)(AAG)(CAG)(CAA)(CAG)$_{30}$													4										1																					5
45	(CAG)$_{2}$(CAA)(AAG)(CAG)(CAA)(CAG)$_{39}$					2																																							2
49	(CAG)$_{2}$(CAA)(AAG)(CAG)(CAA)(CAG)$_{43}$																								1																				1
56	(CAG)$_{2}$(CAA)(AAG)(CAG)(CAA)(CAG)$_{50}$		1																1																										2
58	(CAG)$_{2}$(CAA)(AAG)(CAG)(CAA)(CAG)$_{52}$																1																												1
59	(CAG)$_{2}$(CAA)(AAG)(CAG)(CAA)(CAG)$_{53}$																																	1												1
60	(CAG)$_{2}$(CAA)(AAG)(CAG)(CAA)(CAG)(CAA)(CAG)$_{52}$																							1																					1
60	(CAG)$_{2}$(CAA)(AAG)(CAG)(CAA)(CAG)$_{54}$									1																															1	1				3
61	(CAG)$_{2}$(CAA)(AAG)(CAG)$_{57}$																	1																											1
61	(CAG)$_{2}$(CAA)(AAG)(CAG)(CAA)(CAG)$_{55}$									2										1				3																1						7
62	(CAG)$_{2}$(CAA)(AAG)(CAG)$_{58}$																	3																											3
62	(CAG)$_{2}$(CAA)(AAG)(CAG)(CAA)(CAG)$_{2}$(CAA)(CAG)$_{53}$																			1																									1
62	(CAG)$_{2}$(CAA)(AAG)(CAG)(CAA)(CAG)$_{56}$									1				2						1				1										2						1	2	1				11
63	(CAG)$_{63}$													1																															1
63	(CAG)$_{2}$(CAA)(AAG)(CAG)$_{59}$					1												1																											2
63	(CAG)$_{2}$(CAA)(AAG)(CAG)(CAA)(CAG)$_{57}$													2						2				2										2					1	2	2	4				17
64	(CAG)$_{2}$(CAA)(AAG)(CAG)(CAA)(CAG)$_{58}$													1																		1	1	1					4	2	4	2		1		17
65	(CAG)$_{2}$(CAA)(AAG)(CAG)(CAA)(CAG)$_{59}$																			1												2	1	2						1	1					8
66	(CAG)$_{2}$(CAA)(AAG)(CAG)(CAA)(CAG)$_{60}$																					1										4	1	1							1				8
67	(CAG)$_{2}$(CAA)(AAG)(CAG)$_{63}$								2																																						2
67	(CAG)$_{2}$(CAA)(AAG)(CAG)(CAA)(CAG)$_{61}$	7										1			1					1	2	5	1																						1	19
68	(CAG)$_{2}$(CAA)(AAG)(CAG)$_{64}$								2																																						2
68	(CAG)$_{2}$(CAA)(AAG)(CAG)(CAA)(CAG)$_{62}$	1				1		1				1								1		1																									6
69	(CAG)$_{2}$(CAA)(AAG)(CAG)$_{65}$								1																																						1
69	(CAG)$_{2}$(CAA)(AAG)(CAG)(CAA)(CAG)$_{63}$	1				1					1	2	1								3				1	4									2										1	17
70	(CAG)$_{2}$(CAA)(AAG)(CAG)(CAA)(CAG)$_{64}$					1								2		2	1			1					1		1	1					1				3	1	3						1	1	20
71	(CAG)$_{2}$(CAA)(CAG)$_{68}$									1																																			1
71	(CAG)$_{2}$(CAA)(AAG)(CAG)(CAA)(CAG)(CAA)(CAG)$_{63}$																								1																				1
71	(CAG)$_{2}$(CAA)(AAG)(CAG)(CAA)(CAG)$_{65}$				1	4		1								3								2		1		1	1			1				2	1	2										20
72	(CAG)$_{2}$(CAA)(AAG)(CAG)(CAA)(CAG)$_{2}$(CAA)(CAG)$_{63}$										1																																		1
72	(CAG)$_{2}$(CAA)(AAG)(CAG)(CAA)(CAG)(CAA)(CAG)$_{64}$									1																																			1
72	(CAG)$_{2}$(CAA)(AAG)(CAG)(CAA)(CAG)$_{66}$				1	1						3				2	3										1		1				3					3	2	1									21
73	(CAG)$_{2}$(CAA)(CAG)$_{2}$(CAA)(CAG)$_{67}$									1																1																			2
73	(CAG)$_{2}$(CAA)(AAG)(CAG)(CAA)(CAG)$_{67}$		1	2				1			1								3								2			2								1	1								1	15
73	(CAG)$_{2}$(CAA)(AAG)(CAG)$_{69}$																															1														1
74	(CAG)$_{2}$(CAA)(AAG)(CAG)(CAA)(CAG)$_{18}$(CAA)(CAG)$_{49}$																														1																1
74	(CAG)$_{2}$(CAA)(AAG)(CAG)(CAA)(CAG)$_{3}$(CAA)(CAG)$_{64}$																												1																		1
74	(CAG)$_{2}$(CAA)(AAG)(CAG)(CAA)(CAG)$_{68}$			7	1								1			1			2								1	1		1		3				1	1							4		24
75	(CAG)$_{2}$(CAA)(AAG)(CAG)(CAA)(CAG)$_{69}$		1	2				1			1								3										1	2	1					1		1								2	16
76	(CAG)$_{2}$(CAA)(AAG)(CAG)(CAA)(CAG)$_{5}$(CAA)(CAG)$_{64}$																												1																		1
76	(CAG)$_{2}$(CAA)(AAG)(CAG)(CAA)(CAG)$_{70}$							1									2												1																		4
77	(CAG)$_{2}$(CAA)(AAG)(CAG)(CAA)(CAG)$_{71}$		2														4												1																		7
78	(CAG)$_{2}$(CAA)(AAG)(CAG)$_{74}$																																										1			1
78	(CAG)$_{2}$(CAA)(AAG)(CAG)(CAA)(CAG)$_{72}$		2														1																												3
79	(CAG)$_{2}$(CAA)(AAG)(CAG)(CAA)(CAG)$_{73}$																1										1																			2
80	(CAG)$_{2}$(CAA)(AAG)(CAG)(CAA)(CAG)$_{74}$						2										1																												3
81	(CAG)$_{2}$(CAA)(AAG)(CAG)$_{77}$																																										2			2
82	(CAG)$_{2}$(CAA)(AAG)(CAG)$_{78}$																																										1			1
82	(CAG)$_{2}$(CAA)(AAG)(CAG)(CAA)(CAG)$_{76}$						1																																						1
83	(CAG)$_{2}$(CAA)(AAG)(CAG)$_{79}$																																										2			2
84	(CAG)$_{2}$(CAA)(AAG)(CAG)$_{80}$																																										1			1
85	(CAG)$_{2}$(CAA)(AAG)(CAG)$_{81}$																																										1			1
86	(CAG)$_{2}$(CAA)(AAG)(CAG)$_{82}$																																										1			1
Total		18	15	23	9	21	9	10	10	13	12	9	9	12	12	12	10	7	15	14	7	10	5	10	10	8	8	3	7	9	7	8	6	10	8	9	9	4	5	10	12	10	10	7	8	440

Table S1 ***The number of clones with a particular sequence detected for each participant in the "Cloning" sub-cohort.*** *The* ***bold*** *line indicates the cut-off between non-expanded (≤49 repeats) and expanded (≥56 repeats). Clones with a loss of canonical interruptions are highlighted in red, while those with additional interruptions are highlighted in green.*

Repeat Size	Repeat Tract Sequence	Participant																																											Overall Percentage
		1	2	3	4	5	6	7	8	9	10	11	12	13	14	15	16	17	18	19	20	21	22	23	24	25	26	27	28	29	30	31	32	33	34	35	36	37	38	39	40	41	42	43	44	
13	(CAG)$_2$(CAA)(AAG)(CAG)(CAA)(CAG)$_{7}$																			7.1																										0.2
14	(CAG)$_2$(CAA)(AAG)(CAG)(CAA)(CAG)$_{8}$														33.3			28.6		28.6		30.0																								3.0
18	(CAG)$_2$(CAA)(AAG)(CAG)(CAA)(CAG)$_{12}$																																	10.0												0.2
19	(CAG)$_2$(CAA)(AAG)(CAG)(CAA)(CAG)$_{13}$		6.7																										28.6																	0.7
21	(CAG)$_2$(CAA)(AAG)(CAG)(CAA)(CAG)$_{15}$	5.6								7.7		11.1	11.1								28.6																	25.0								1.6
22	(CAG)$_2$(CAA)(AAG)(CAG)(CAA)(CAG)$_{16}$	5.6										44.4	44.4						6.7											11.1												10.0				2.7
23	(CAG)$_2$(CAA)(AAG)(CAG)(CAA)(CAG)$_{17}$	38.9				9.5				38.5									33.3				20.0	10.0	40.0		37.5			33.3											16.7					7.5
24	(CAG)$_2$(CAA)(AAG)(CAG)(CAA)(CAG)$_{18}$				66.7	38.1																																		30.0						3.9
25	(CAG)$_2$(CAA)(AAG)(CAG)(CAA)(CAG)$_{19}$						11.1																																							0.2
27	(CAG)$_2$(CAA)(AAG)(CAG)(CAA)(CAG)$_{21}$						55.6				41.7																									22.2										2.7
28	(CAG)$_2$(CAA)(AAG)(CAG)(CAA)(CAG)$_{22}$																									12.5																				0.2
29	(CAG)$_2$(CAA)(AAG)(CAG)(CAA)(CAG)$_{23}$								50.0							8.3										12.5																				1.6
30	(CAG)$_2$(CAA)(AAG)(CAG)(CAA)(CAG)$_{24}$							50.0								50.0																											10.0			2.7
31	(CAG)$_2$(CAA)(AAG)(CAG)(CAA)(CAG)$_{25}$		6.7																																											0.2
32	(CAG)$_2$(CAA)(AAG)(CAG)(CAA)(CAG)$_{26}$		13.3	13.0																																										1.1
33	(CAG)$_2$(CAA)(AAG)(CAG)(CAA)(CAG)$_{27}$		20.0	39.1										8.3																		12.5												14.3	25.0	3.9
34	(CAG)$_2$(CAA)(AAG)(CAG)(CAA)(CAG)$_{28}$		6.7																					10.0																						0.5
35	(CAG)$_2$(CAA)(AAG)(CAG)(CAA)(CAG)$_{29}$													8.3																																0.2
36	(CAG)$_2$(CAA)(AAG)(CAG)(CAA)(CAG)$_{30}$													33.3										10.0																						1.1
45	(CAG)$_2$(CAA)(AAG)(CAG)(CAA)(CAG)$_{39}$					9.5																																								0.5
49	(CAG)$_2$(CAA)(AAG)(CAG)(CAA)(CAG)$_{43}$																								10.0																					0.2
56	(CAG)$_2$(CAA)(AAG)(CAG)(CAA)(CAG)$_{50}$		6.7																	7.1																										0.5
58	(CAG)$_2$(CAA)(AAG)(CAG)(CAA)(CAG)$_{52}$																10.0																													0.2
59	(CAG)$_2$(CAA)(AAG)(CAG)(CAA)(CAG)$_{53}$																																	10.0												0.2
60	(CAG)$_2$(CAA)(AAG)(CAG)(CAA)(CAG)(CAA)(CAG)$_{52}$																							10.0																						0.2
60	(CAG)$_2$(CAA)(AAG)(CAG)(CAA)(CAG)$_{54}$									7.7																															8.3	10.0				0.7
61	(CAG)$_2$(CAA)(AAG)(CAG)$_{57}$																	14.3																												0.2
61	(CAG)$_2$(CAA)(AAG)(CAG)(CAA)(CAG)$_{55}$									15.4										7.1				30.0																10.0						1.6
62	(CAG)$_2$(CAA)(AAG)(CAG)$_{58}$																	42.9																												0.7
62	(CAG)$_2$(CAA)(AAG)(CAG)(CAA)(CAG)$_2$(CAA)(CAG)$_{53}$																			7.1																										0.2
62	(CAG)$_2$(CAA)(AAG)(CAG)(CAA)(CAG)$_{56}$									7.7				16.7						7.1				10.0										20.0						10.0	16.7	10.0				2.5
63	(CAG)$_{63}$													8.3																																0.2
63	(CAG)$_2$(CAA)(AAG)(CAG)$_{59}$					4.8												14.3																												0.5
63	(CAG)$_2$(CAA)(AAG)(CAG)(CAA)(CAG)$_{57}$													16.7						14.3				20.0										20.0					20.0	20.0	16.7	40.0				3.9
64	(CAG)$_2$(CAA)(AAG)(CAG)(CAA)(CAG)$_{58}$													8.3																		12.5	16.7	10.0					80.0	20.0	33.3	20.0		14.3		3.9
65	(CAG)$_2$(CAA)(AAG)(CAG)(CAA)(CAG)$_{59}$																			7.1												25.0	16.7	20.0						10.0	8.3					1.8
66	(CAG)$_2$(CAA)(AAG)(CAG)(CAA)(CAG)$_{60}$																					10.0											66.7	10.0	12.5							10.0				1.8
67	(CAG)$_2$(CAA)(AAG)(CAG)$_{63}$								20.0																																					0.5
67	(CAG)$_2$(CAA)(AAG)(CAG)(CAA)(CAG)$_{61}$	38.9										11.1			8.3					7.1	28.6	50.0	20.0																						12.5	4.3
68	(CAG)$_2$(CAA)(AAG)(CAG)$_{64}$								20.0																																					0.5
68	(CAG)$_2$(CAA)(AAG)(CAG)(CAA)(CAG)$_{62}$	5.6				4.8		10.0				11.1								7.1		10.0																								1.4
69	(CAG)$_2$(CAA)(AAG)(CAG)$_{65}$								10.0																																					0.2
69	(CAG)$_2$(CAA)(AAG)(CAG)(CAA)(CAG)$_{63}$	5.6				4.8					8.3	22.2	11.1								42.9				10.0	50.0									25.0										12.5	3.9
70	(CAG)$_2$(CAA)(AAG)(CAG)(CAA)(CAG)$_{64}$					4.8							22.2		16.7	8.3			6.7				20.0		10.0	12.5					14.3				37.5	11.1	33.3							14.3	12.5	4.5
71	(CAG)$_2$(CAA)(CAG)$_{68}$									7.7																																				0.2
71	(CAG)$_2$(CAA)(AAG)(CAG)(CAA)(CAG)(CAA)(CAG)$_{63}$																								10.0																					0.2
71	(CAG)$_2$(CAA)(AAG)(CAG)(CAA)(CAG)$_{65}$				11.1	19.0		10.0							25.0								40.0		10.0		12.5	33.3			14.3				25.0	11.1	22.2									4.5
72	(CAG)$_2$(CAA)(AAG)(CAG)(CAA)(CAG)$_2$(CAA)(CAG)$_{63}$										8.3																																			0.2
72	(CAG)$_2$(CAA)(AAG)(CAG)(CAA)(CAG)(CAA)(CAG)$_{64}$									7.7																																				0.2
72	(CAG)$_2$(CAA)(AAG)(CAG)(CAA)(CAG)$_{66}$				11.1	4.8					25.0				16.7	25.0									10.0		12.5				42.9					33.3	22.2	25.0								4.8
73	(CAG)$_2$(CAA)(CAG)$_2$(CAA)(CAG)$_{67}$									7.7																12.5																				0.5
73	(CAG)$_2$(CAA)(AAG)(CAG)(CAA)(CAG)$_{67}$		6.7	8.7				10.0			8.3								20.0								25.0			22.2							11.1	25.0							12.5	3.4
73	(CAG)$_2$(CAA)(AAG)(CAG)$_{69}$																															12.5														0.2
74	(CAG)$_2$(CAA)(AAG)(CAG)(CAA)(CAG)$_{18}$(CAA)(CAG)$_{49}$																														14.3															0.2
74	(CAG)$_2$(CAA)(AAG)(CAG)(CAA)(CAG)$_3$(CAA)(CAG)$_{64}$																												14.3																	0.2
74	(CAG)$_2$(CAA)(AAG)(CAG)(CAA)(CAG)$_{68}$			30.4	11.1								11.1			8.3			13.3								12.5	33.3		11.1		37.5				11.1	11.1							57.1		5.5
75	(CAG)$_2$(CAA)(AAG)(CAG)(CAA)(CAG)$_{69}$		6.7	8.7				10.0			8.3								20.0										14.3	22.2	14.3					11.1		25.0							25.0	3.6
76	(CAG)$_2$(CAA)(AAG)(CAG)(CAA)(CAG)$_5$(CAA)(CAG)$_{64}$																												14.3																	0.2
76	(CAG)$_2$(CAA)(AAG)(CAG)(CAA)(CAG)$_{70}$							10.0									20.0												14.3																	0.9
77	(CAG)$_2$(CAA)(AAG)(CAG)(CAA)(CAG)$_{71}$		13.3														40.0												14.3																	1.6
78	(CAG)$_2$(CAA)(AAG)(CAG)$_{74}$																																										10.0			0.2
78	(CAG)$_2$(CAA)(AAG)(CAG)(CAA)(CAG)$_{72}$		13.3														10.0																													0.7
79	(CAG)$_2$(CAA)(AAG)(CAG)(CAA)(CAG)$_{73}$																10.0											33.3																		0.5
80	(CAG)$_2$(CAA)(AAG)(CAG)(CAA)(CAG)$_{74}$						22.2										10.0																													0.7
81	(CAG)$_2$(CAA)(AAG)(CAG)$_{77}$																																										20.0			0.5
82	(CAG)$_2$(CAA)(AAG)(CAG)$_{78}$																																										10.0			0.2
82	(CAG)$_2$(CAA)(AAG)(CAG)(CAA)(CAG)$_{76}$						11.1																																							0.2
83	(CAG)$_2$(CAA)(AAG)(CAG)$_{79}$																																										20.0			0.5
84	(CAG)$_2$(CAA)(AAG)(CAG)$_{80}$																																										10.0			0.2
85	(CAG)$_2$(CAA)(AAG)(CAG)$_{81}$																																										10.0			0.2
86	(CAG)$_2$(CAA)(AAG)(CAG)$_{82}$																																										10.0			0.2
**Overall Percentage**		4.1	3.4	5.2	2.0	4.8	2.0	2.3	2.3	3.0	2.7	2.0	2.0	2.7	2.7	2.7	2.3	1.6	3.4	3.2	1.6	2.3	1.1	2.3	2.3	1.8	1.8	0.7	1.6	2.0	1.6	1.8	1.4	2.3	1.8	2.0	2.0	0.9	1.1	2.3	2.7	2.3	2.3	1.6	1.8	100.0

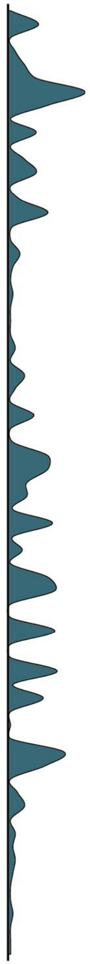

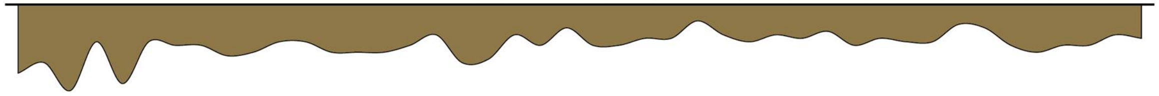

Table S2 **Frequency of each clone sequence for each participant as a percentage of total clones for that participant.** This corrects for the clone depth sequenced for each participant. The composition of the total clone population based on repeat tract sequence is shown in the right-hand column with a schematic graph depicting the frequency distribution in blue. Each participant's contribution to the total clone population is shown in the last row with a schematic graph depicting the frequency distribution in brown. The **bold** line indicates the cut-off between non-expanded (≤49 repeats) and expanded (≥56 repeats). Clones with a loss of canonical interruptions are highlighted in red, while those with additional interruptions are highlighted in green.

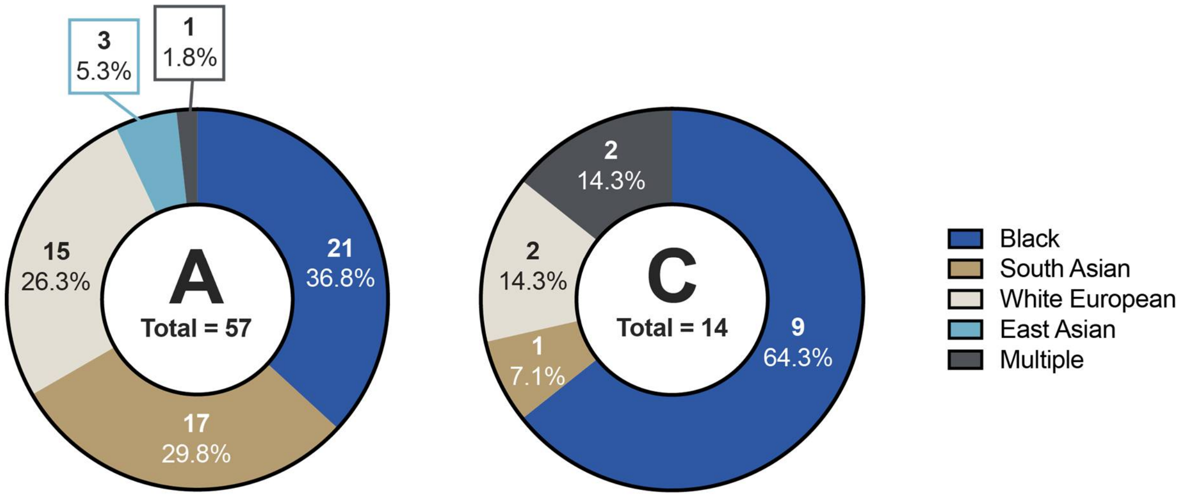

Figure S1 ***Pie charts showing the ethnic distribution of the expanded allele rs7158733 SNP variant.*** *Differences between participants' ethnicity and the presence of the A^{1118} or C^{1118} rs7158733 SNP expanded allele variant were not statistically significant (χ^2 = 9.88; df = 4; Fisher's exact test, p = 0.054).*

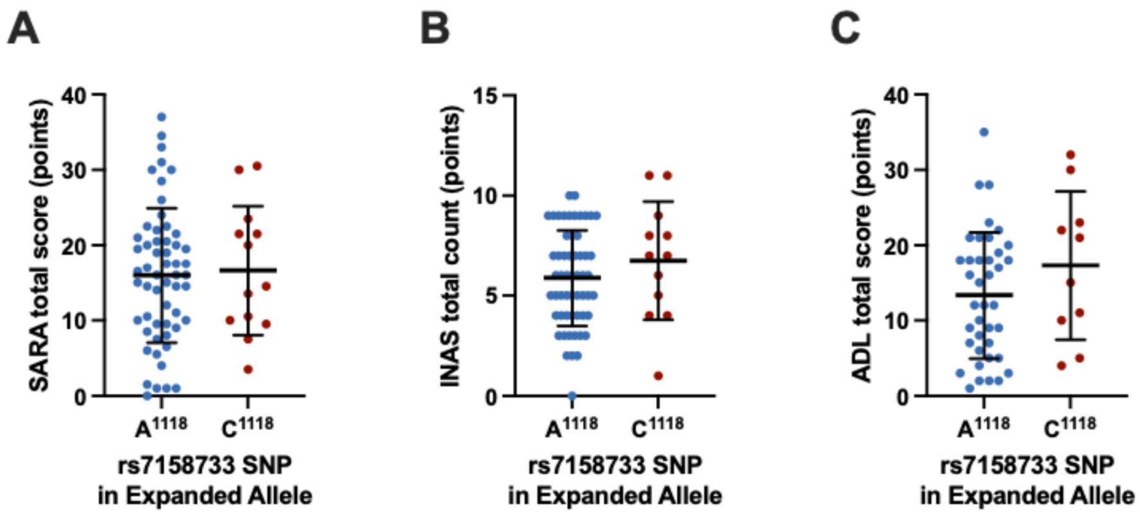

Figure S2 ***Scatterplots comparing the baseline patient rating scales of the SCA3/MJD subjects based on expanded allele rs7158733 SNP variant.*** *(**A**) There was no significant difference in the means of total SARA scores between participants with the A^{1118} SNP expanded allele (n = 56) and those with the C^{1118} SNP expanded allele (n = 13) (Unpaired two-tailed t-test, p = 0.817). (**B**) There was no significant difference in the means of total INAS counts between participants with the A^{1118} SNP expanded allele (n = 56) and those with the C^{1118} SNP expanded allele (n = 12) (Unpaired two-tailed t-test, p = 0.275). (**C**) There was also no significant difference in the means of total ADL scores between participants with the A^{1118} SNP expanded allele (n = 39) and those with the C^{1118} SNP expanded allele (n = 10) (Unpaired two-tailed t-test, p = 0.204). Means are shown with the bold line with error bars showing the standard deviation.*

Symptom at onset	A^{1118} (n = 41)	C^{1118} (n = 14)	P-value[a]
Ataxia	39 (95.1)	13 (92.9)	1.000
Parkinsonism	1 (2.4)	0 (0.0)	1.000
Dystonia	0 (0.0)	0 (0.0)	N/A
Spasticity	1 (2.4)	1 (7.1)	0.448
Peripheral neuropathy	0 (0.0)	0 (0.0)	N/A

Table S3 ***Symptoms at the onset of the disease in the SCA3/MJD subjects based on rs7158733 SNP genotype of their expanded alleles.*** *Data is presented as the number of patients and the groupwise percentage of patients. N/A: not applicable. [a]Fisher's exact test.*

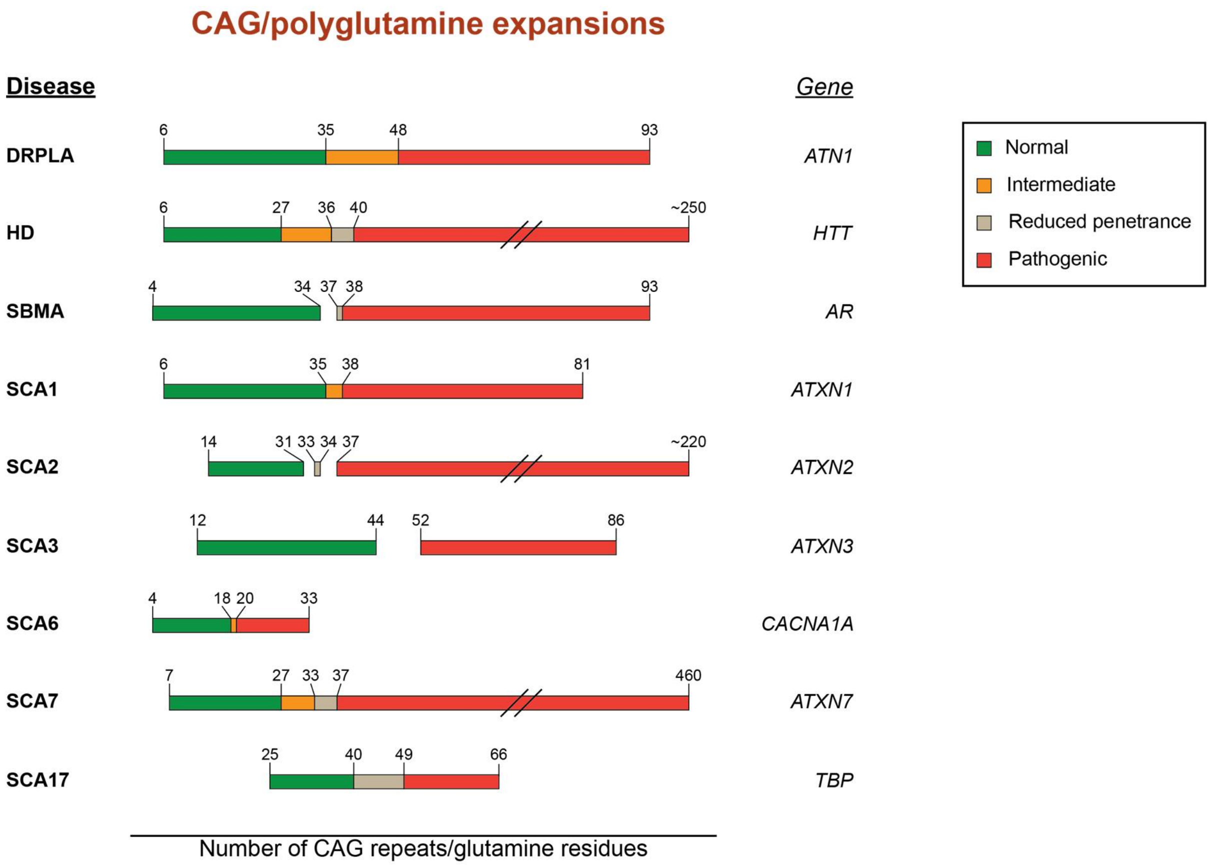

Figure S3 ***Repeat ranges for polyglutamine CAG repeat disorders.*** *Each disease is shown in* ***bold*** *on the left, and the corresponding affected gene is shown in italics on the right. Normal repeat sizes are depicted in green blocks, intermediate repeat sizes are depicted in orange blocks, reduced penetrance repeat sizes are depicted in taupe, and pathogenic repeat sizes are depicted in red. Intermediate alleles of 27-33 repeats in* ATXN2 *and ≥32 in* ATXN1 *have been considered a risk factor for amyotrophic lateral sclerosis (ALS)*[1, 2]*. However, this figure focuses on relating repeat length to the appropriate polyglutamine disorder. SBMA, SCA2, and SCA3 are the only polyglutamine diseases with a gap between normal allele sizes and alleles in the reduced penetrance or pathogenic range, according to reports to date. Abbreviations: DRPLA, dentatorubral-pallidoluysian atrophy; HD, Huntington's disease; SCA, spinocerebellar ataxia. Data was compiled predominantly from* http://genereviews.org *(accession date 18th March 2025).*

References

1. Borghero G, Pugliatti M, Marrosu F, Marrosu MG, Murru MR, Floris G, *et al.* ATXN2 is a modifier of phenotype in ALS patients of Sardinian ancestry. *Neurobiology of aging* 2015, **36**(10)**:** 2906.e2901–2905.

2. Conforti FL, Spataro R, Sproviero W, Mazzei R, Cavalcanti F, Condino F, *et al.* Ataxin-1 and ataxin-2 intermediate-length PolyQ expansions in amyotrophic lateral sclerosis. *Neurology* 2012, **79**(24)**:** 2315–2320.
